# Sequencing the USDA core soybean collection reveals gene loss during domestication and breeding

**DOI:** 10.1002/tpg2.20109

**Published:** 2021-06-24

**Authors:** Philipp E. Bayer, Babu Valliyodan, Haifei Hu, Jacob I. Marsh, Yuxuan Yuan, Tri D. Vuong, Gunvant Patil, Qijian Song, Jacqueline Batley, Rajeev K. Varshney, Hon‐Ming Lam, David Edwards, Henry T. Nguyen

**Affiliations:** ^1^ School of Biological Sciences and Inst. of Agriculture The Univ. of Western Australia Crawley WA Australia; ^2^ Dep. of Agriculture and Environmental Sciences Lincoln Univ. Jefferson, City MO 65101 USA; ^3^ Div. of Plant Sciences and National Ctr. for Soybean Biotechnology Univ. of Missouri Columbia MO USA; ^4^ Ctr. for Soybean Research of the State Key Lab. of Agrobiotechnology and School of Life Sciences The Chinese Univ. of Hong Kong Shatin Hong Kong China; ^5^ Dep. of Plant and Soil Science Texas Tech Univ. Lubbock TX USA; ^6^ U.S. Dep. of Agriculture–Agricultural Research Service Soybean Genomics and Improvement Lab. Beltsville MD USA; ^7^ Ctr. of Excellence in Genomics & Systems Biology International Crops Research Inst. for the Semi‐Arid Tropics (ICRISAT) Patancheru India; ^8^ State Agricultural Biotechnology Ctr., Crop Research Innovation Ctr., Food Futures Inst. Murdoch Univ. Murdoch WA Australia

## Abstract

The gene content of plants varies between individuals of the same species due to gene presence/absence variation, and selection can alter the frequency of specific genes in a population. Selection during domestication and breeding will modify the genomic landscape, though the nature of these modifications is only understood for specific genes or on a more general level (e.g., by a loss of genetic diversity). Here we have assembled and analyzed a soybean (*Glycine* spp.) pangenome representing more than 1,000 soybean accessions derived from the USDA Soybean Germplasm Collection, including both wild and cultivated lineages, to assess genomewide changes in gene and allele frequency during domestication and breeding. We identified 3,765 genes that are absent from the Lee reference genome assembly and assessed the presence/absence of all genes across this population. In addition to a loss of genetic diversity, we found a significant reduction in the average number of protein‐coding genes per individual during domestication and subsequent breeding, though with some genes and allelic variants increasing in frequency associated with selection for agronomic traits. This analysis provides a genomic perspective of domestication and breeding in this important oilseed crop.

## Core Ideas

- We assembled a soybean pangenome based on more than 1,000 lines from the USDA Soybean Germplasm Collection.
- We found 3,765 genes absent from the reference assembly.
- We found a reduction in the number of genes per individual during domestication and breeding.

**Abbreviations**
BSR: brown stem rot
FST: fixation index
GO: gene ontology
LD: linkage disequilibrium
PAV: presence/absence variation
PCA: principal component analyses
QTL: quantitative trait loci
SNPs: single nucleotide polymorphisms
SVs: structural variants

## 1 INTRODUCTION

Cultivated soybean [*Glycine max* (L.) Merr.] is a staple crop that was domesticated 6,000–9,000 years ago in East Asia from wild soybean [*G. soja* (L.) Merr](Carter et al., 2004; Kim et al., 2010), a process that involved a 50% reduction in genetic diversity and the loss of 81% of rare alleles (Hyten et al., 2006; Z. Zhou et al., 2015). Since domestication, diverse cultivated lines have been produced, harboring improved agronomic traits; however, soybean yield is not increasing in pace with the growing demand for this crop (Ray et al., 2013). Soybean production needs to double by 2050 to keep track with a growing population, yet if current yield trends continue, soybean production will grow by only 55% by 2050 (Ray et al., 2013). At the same time, climate change is expected to reduce global soybean yields by 3.1% with each degree Celsius change (C. Zhao et al., 2017).

Intensive soybean breeding has been associated with further loss of diversity. Around 85% of genes present in North American lines may have been derived from only 19 landraces (Gizlice et al., 1996), and 79% of rare alleles present in diverse landraces have been lost during breeding (Hyten et al., 2006). Genomic analysis of these bottlenecks and the association of the lost diversity with agronomic traits can provide the foundation for increasing diversity in this crop and support the breeding of improved cultivars (Valliyodan et al., 2016).

The increasing availability of crop genome sequence data facilitates the study of genome composition changes during domestication and breeding. While many studies have examined the diversity of single nucleotide polymorphisms (SNPs) in populations, there has been increasing acknowledgment of the importance of gene presence/absence variation (PAV) in crop species (Alonge et al., 2020; Gao et al., 2019; Hurgobin & Edwards, 2017; Li et al., 2014; Liu et al., 2020; Lu et al., 2015), leading to the growth of pangenomics (Bayer et al., 2020; Danilevicz et al., 2020; Golicz et al., 2016; Golicz et al., 2020). Pangenomes have been constructed for several crop species, including maize (*Zea mays* L.; Hirsch et al., 2014), *Brassica oleracea* L. (Golicz et al., 2016), wheat (*Triticum aestivum* L.; Montenegro et al., 2017), canola (*Brassica napus* L.; Hurgobin et al., 2018), sesame (*Sesamum indicum* L.; Yu et al., 2019a), tomato (*Solanum lycopersicum* L.; Gao et al., 2019), rice (*Oryza sativa* L; Q. Zhao et al., 2018; Y. Zhou et al., 2020), and pigeon pea (*Cajanus cajan* L.; J. Zhao et al., 2020). These studies used whole‐genome resequencing to assemble genomic regions not present in the reference genomes and to call gene PAV. They found extensive gene PAV ranging from 19% of genes being dispensable in *B. oleracea* (Golicz et al., 2016) to almost 40% of genes being dispensable in hexaploid bread wheat (*Triticum aestivum* L.; Montenegro et al., 2017), and in almost all of these studies, dispensable genes were enriched for biotic and abiotic stress‐related annotations.

**Core Ideas**

- We assembled a soybean pangenome based on more than 1,000 lines from the USDA Soybean Germplasm Collection.
- We found 3,765 genes absent from the reference assembly.
- We found a reduction in the number of genes per individual during domestication and breeding.

Comparative genomics methods have been applied to soybean. A comparison of seven whole‐genome assemblies of wild *G. soja* lines found loss‐of‐function frameshifts in domestication‐related genes and PAV‐regions reduced in frequency in *G. max* compared with *G. soja* (Li et al., 2014). A subsequent study examined 302 wild and cultivated soybean genomes to investigate the impact of domestication (Z. Zhou et al., 2015). This study identified 10 genomic regions under selection linked to nine domestication or breeding traits, mostly associated with oil content and fatty acid biosynthesis. A later study across 106 U.S. soybean lines identified 146 regions under selection (Valliyodan et al., 2016). They found that 43% of SNPs and 50% of PAV regions were not shared between wild *G. soja* and cultivated lines. Together these studies highlight the impact of domestication on the *Glycine* genome.

A recent soybean pangenome compares 26 de novo genome assemblies and data from an additional 2,872 wild, landrace, and cultivated lines (Liu et al., 2020). They identify 55,402 structural variants (SVs), with wild soybeans containing more SVs than landraces and cultivars. Genes were grouped into gene families, and only 35.88% of gene families were present in all lines. As with other pangenomes, dispensable genes were enriched with annotations for defense response, while core genes were associated with metabolic pathways. A genome‐wide association study using these SVs as input identified a 10‐kb deletion around a hydrophobic protein gene associated with seed luster, highlighting the importance of PAV in selection. This study also identified domestication‐related SVs, including a 360‐kb inversion on chromosome 7 that occurred approximately 4,700 years ago during soybean domestication.

Modern U.S. soybean breeding has led to a yield increase of 29 kg ha^−1^ yr^−1^ (Rincker et al., 2014), and understanding the genomic basis behind this improvement may provide indicators for further soybean improvement and adaptation. To investigate this, we assembled a pangenome and examined gene PAV as well as SNP diversity across 1,110 soybean lines (157 wild *G. soja*, 723 landraces, 228 cultivars, and two unclassified lines). These include 886 newly sequenced individuals, which represent the diversity present in the USDA Soybean Germplasm Collection. We demonstrate both a reduction in genetic diversity and a contraction in both gene number and estimated genome size during both during domestication and the subsequent breeding of modern U.S. soybean lines.

## 2 MATERIALS AND METHODS

### 2.1 Data availability

The sequence metadata for all 1,110 soybean accessions are summarized in Supplemental Table S2. Of these lines, 118 lines were previously published in PRJNA257011 (Fang et al., 2017; Z. Zhou et al., 2015) and 104 lines were previously published in PRJNA289660 (Valliyodan et al., 2016). All newly sequenced data are publicly available from the SRA project PRJNA639876. The assembled genomes and other data are available in Bayer et al. (Bayer et al., 2020). The constructed pangenome can be visualized using JBrowse (Buels et al., 2016) at http://appliedbioinformatics.com.au/soybean/. Pangenome annotation used available RNA‐seq from NCBI (PRJNA238008,PRJNA246058 PRJNA246315,PRJNA246314,PRJNA246783,PRJNA197251,PRJNA254333,PRJNA280872,PRJNA149185,PRJNA350330,PRJNA304631,PRJNA389558,PRJNA182292,PRJNA197251). Protein sequences for *G. max, G. soja, Medicago truncatula* Gaertn.*, Vigna radiata* (L.) R. Wilczek var. *radiata*, and *Vigna angularis* (Willd.) Ohwi & H. Ohashi var. *angularis* were downloaded from Soybase (https://soybase.org/data/public/).

### 2.2 Soybean germplasm, DNA isolation and sequencing

Diverse soybean germplasm were selected from the USDA Soybean Germplasm Collection (Song et al., 2015) and the seeds were germinated in the University of Missouri greenhouse for leaf sample collection and DNA extraction. Total DNA was extracted using the cetyltrimethylammonium bromidemethod (Murray & Thompson, 1980), and the sample heterogeneity was tested using Illumina Infinium SoySNP6K BeadChips BARCSoySNP6K beadchips containing SNPs that were selected from SoySNP50K (Song et al., 2013). All sequencing libraries were constructed using 5 μg of genomic DNA from each soybean germplasm following the Illumina sequencing protocols (Illumina Inc.). Paired‐end sequencing libraries with an insert size of ∼300 bp were sequenced on an Illumina HiSeq 2000 sequencer, at a minimum depth of 8.5× genome equivalent. Germplasm details, sequencing depth, and sequence identifiers are presented in Supplemental Table S1.

### 2.3 Pangenome construction

The pangenome was assembled using a previously published pipeline (Golicz et al., 2016) using the chromosome‐level Lee soybean assembly as the starting reference (Valliyodan et al., 2019). The pipeline consists of steps to assemble reads that do not align with the reference. The chloroplast and mitochondrial genomes (NC_020455.1; NC_007942.1) were first added to the reference. Adapters were removed from the sequence reads using Trimmomatic (Bolger et al., 2014) v0.36, and reads were aligned with the reference using Bowtie2 (Langmead & Salzberg, 2012) v2.3.3.1 (–end‐to‐end –sensitive ‐I 0 ‐X 1000). Unaligned reads were assembled using MaSuRCA (Zimin et al., 2013) v3.3.1, and contigs greater than 500 bp were retained.

### 2.4 Annotation of the soybean pangenome

The pangenome was annotated using Augustus (Stanke et al., 2006) and SNAP (Korf, 2004). RNA‐Seq data was trimmed to remove the low‐quality sequences and adapters removed using Trimmomatic (Bolger, Lohse & Usadel, 2014) v0.36. The clean reads were mapped to the pangenome using Hisat2 (Kim et al., 2015) v2.1.0 and used to construct gene models using StringTie (Pertea et al., 2015) v2.0 and TransDecoder (Haas et al., 2013) v5.5.0. The Soybase protein sequences for *G. max, G. soja, M. truncatula, V. radiata* var. *radiata*, and *V. angularis* var. *angularis* were clustered and redundant sequences removed using CD‐HIT (W. Li & Godzik, 2006) v4.6.8 with default settings. The de novo predicted and evidence models were used to annotate the pangenome using Maker 2 with the clustered Soybase proteins as external evidence (Holt & Yandell, 2011). Repeats were masked using RepeatMasker v4.0.4 (Smit & Hubley, 2008) using all repeats stored in Repbase 20150807 (Jurka et al., 2005). Predicted genes with protein length shorter than 33 amino acids were removed.

### 2.5 PAV analysis

Genomic reads for each accession were aligned to the pangenome using Bowtie2 (Langmead & Salzberg, 2012) v2.3.3.1 (–end‐to‐end –sensitive ‐I 0 ‐X 1000). A gene is considered as missing when the horizontal coverage across exons is less than 5% and the vertical coverage less than two times as used in SGSGeneLoss (Golicz et al., 2016; Golicz et al., 2015) using Mosdepth v0.2.6 (Pedersen & Quinlan, 2018). A PAV matrix was generated showing the presence or absence of each gene for each accession. Statistical significance of gene frequency changes due to selection during domestication or breeding was calculated using Fisher's exact test. *P*‐values were adjusted for multiple comparisons using the Bonferroni method as implemented in p.adjust from R v3.5.0 (R Core Team, 2020). Genes with an adjusted *p*‐value < .001 and difference frequency between groups ≥10% were identified.

Genome sizes were estimated using JELLYFISH v2.2.6 (settings: ‐h 1,000,000 for the upper limit of the histogram; Marcais & Kingsford, 2011) and GenomeScope (Vurture et al., 2017). Genome size estimates with model fits below 95% were removed, as were extreme outlier estimates (below 900 Mb, above 1,200 Mb).

### 2.6 GO analysis

Functional annotation was performed using Blast2GO (Conesa et al., 2005) v2.5. Genes were aligned to the proteins in the Viridiplantae database using BLASTP (Camacho et al., 2009; E‐values <1 × 10‐5). Gene ontology (GO) analysis was conducted using topGO (Alexa & Rahnenführer, 2009) and Fisher's exact test with ‘elim’ used to correct for multiple comparisons.

### 2.7 SNP discovery and population genetics analysis

Clean reads were mapped to the pangenome using BWA‐MEM (H. Li, 2013) v0.7.17 with default settings and duplicates removed by Picard tools (http://broadinstitute.github.io/picard/). Reads were realigned by GATK (McKenna et al., 2010) v3.8‐1‐0 RealignerTargetCreator and IndelRealigner, followed by variant calling using GATK HaplotypeCaller. The resulting SNPs were filtered (QD < 2.0 || MQ < 40.0 || FS > 60.0 || QUAL < 60.0 || MQrankSum < −12.5 || ReadPosRankSum < −8.0) to remove low‐quality SNPs.

High‐confidence SNPs were identified by removing SNPs with minor allele frequency <0.05 and missing genotype rate <10% using VCFtools (Danecek et al., 2011). Neighbor‐joining phylogenetic trees were constructed based on PAVs with 1,000 bootstraps using MEGA5 (Tamura et al., 2011). Principal component analysis was performed with the R package logisticPCA (Landgraf & Lee, 2015). Fixation index (FST) values and Tajima's D values were calculated using a 100‐kb sliding window (with a 10‐kb step for FST values calculation) using VCFftools (Danecek et al., 2011). Nucleotide diversity values (π) were calculated using pixy v1.0.4.beta1 using all invariant sites (Korunes & Samuk, 2021). Sliding windows with the top 1% of FST values were selected as significant windows and the overlapped significant windows were merged into the final nonredundant selective regions. The pairwise linkage disequilibrium (LD) between whole genomewide SNPs was calculated for each group based on allele frequency correlations (*r* ^2^) using PopLDdecay (Zhang et al., 2019). Heterozygosity (*F*) was calculated using the –het option in vcftools v0.1.16 (Danecek et al., 2011).

## 3 RESULTS AND DISCUSSION

We have assembled a soybean pangenome and examined the gene content for 1,110 public accessions (886 newly sequenced) from the USDA Soybean Germplasm Collection representing a wide distribution, from the soybean place of origin in East Asia to the current major soybean growing countries (Supplemental Table S1 and Supplemental Figure S1). Accessions were grouped into categories based on breeding history, including 157 wild soybean lineages (*G. soja*), 723 landraces, 228 cultivars, and two unclassified lines (Supplemental Table S1, Supplemental Figure S2 and S3). Cultivars were also split into two groups made up of 46 old cultivars, included cultivars developed during 1910s–1950s and 182 modern cultivars that were developed later. Modern cultivars show increased crop growth rate and produce enhanced yields and yield stability compared with old cultivars (Debruin & Pedersen, 2009). In addition to the Lee genome reference that was used as the basis for pangenome construction (Valliyodan et al., 2019), we assembled an additional 198.4 Mbp of sequence hosting 3,765 high confidence genes (Supplemental Table S2), to produce a pangenome of 1,213 Mbp and 51,414 predicted genes (Supplemental Table S3). Gene PAV was determined for all accessions, which revealed that 86.8% of genes are core (present in all accessions), and the remaining 13.2% are dispensable (absent in at least one accession). The percentage of dispensable genes is lower than previously observed in seven soybean species (∼20% dispensable; Li et al., 2014), which is likely due to differences in gene comparison approaches, as the earlier publication used gene clustering approaches using OrthoMCL, while our study used more stringent read alignment methods. While the read alignment approach for calling PAVs using software such as SGSGeneLoss (Golicz et al., 2015) takes a strict approach in calling a gene as absent, this conservative approach avoids artificially inflating PAV numbers. The proportion of dispensable genes observed here is relatively low compared with some other crop studies that applied read mapping to call PAVs, such as bread wheat (36%; Montenegro et al., 2017), sesame (42%; Yu et al., 2019), or tomato (26%; Gao et al., 2019), although the proportion is similar to pigeon pea (13%; J. Zhao et al., 2020) and rice (11%; Schatz et al., 2014). A recent Chinese soybean pangenome reported 64% of gene families as being dispensable (Liu et al., 2020); however, they did not report the number of individual dispensable genes. The proportion of dispensable genes decreased slightly during domestication from 10.6% of genes in wild soybean to 9.8% in landraces (Supplemental Table S4); however, only 5.9% of genes in modern cultivars are dispensable, reflecting the reduction in diversity during breeding and the fixation of genes.

**FIGURE 1:**
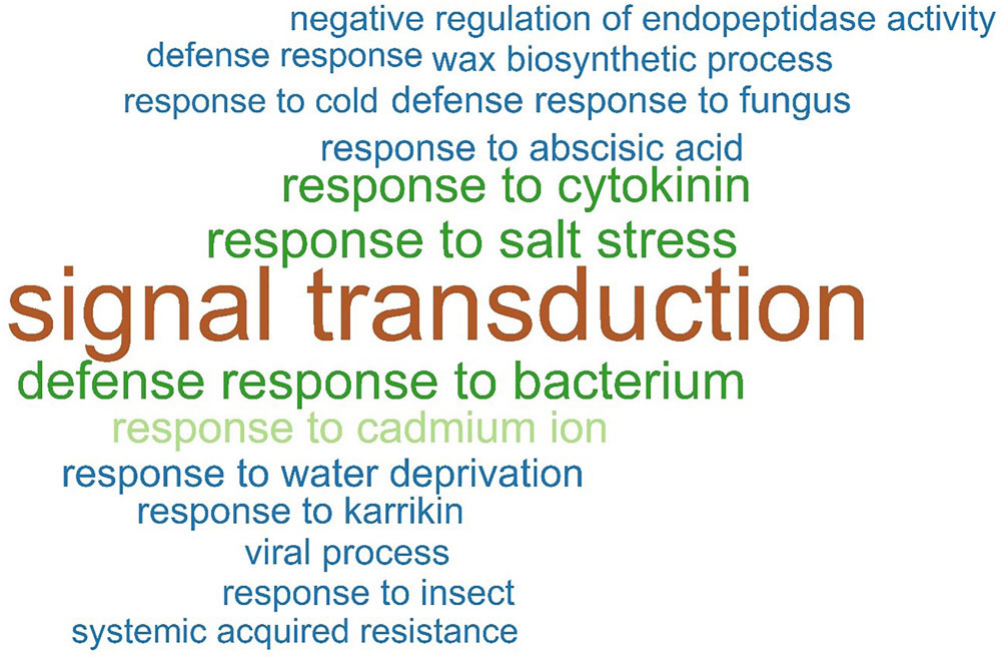
Significantly enriched gene ontology terms among dispensable genes. Font size and color scheme are proportional to –log (p)

Gene ontology analysis suggests that dispensable genes are enriched for terms associated with responses to biotic and abiotic stress, including defense response, response to abscisic acid, and response to salt stress (Figure 1 and Supplemental Table S5). These results are similar to findings in other crop pangenome studies. In soybean, Liu et al. (2020) found GO terms and Pfam domains associated with disease resistance and responses to biotic stimuli. Golicz et al. (2016), observed that *B. oleracea* dispensable genes are enriched for functions associated with disease resistance, response to salt stress, cold, and water deprivation, while (Montenegro et al. 2017) demonstrated that dispensable genes in wheat are enriched for functions associated with response to environmental stress and defense.

Phylogenetic and principal component analyses (PCA) based on gene PAV separated wild lineages and domesticated lines into major clusters, with only a few exceptions (Supplemental Figures S2a, S3). Interestingly, a PCA based on SNPs alone does not divide cultivated lines into subgroups, showing how gene PAV‐based PCA can find patterns not contained in SNPs alone as observed in other plants (De Oliveira et al., 2020; Golicz et al., 2016; Mamidi et al., 2020; Tan et al., 2012; Supplemental Figure 2b). The gene–PAV distribution is similar to that observed by Han et al. (2016) using SNPs, with the domesticated lines forming two clusters, supporting the possibility of multiple domestication events. We find no geographic differences in these two clusters: Domesticated lines of both clusters were collected mostly in Korea (43% of individuals in Cluster 1 and 31% in Cluster 2), followed by Chinese individuals (22 and 29%), Japanese individuals (24 and 25%), and Russian individuals (11 and 14%).

This is contrary to the hypothesis (reviewed in Sedivy et al., 2017) based on domestication‐specific alleles such as the pod shattering‐resistant allele *SHAT1‐5*, which appears in nearly all domesticated soybeans but not in wild soybeans (Dong et al., 2014), or a transposon insertion in a *FLOWERING LOCUS T (FT)* orthologue that appears only in domesticated lines (Wu et al., 2017). However, there is some evidence for multiple domestication events. For example, 302 chloroplast genomes revealed multiple maternal clades indicating that multiple maternal lines were selected in early soybean domestication stages (Fang et al., 2016). Similarly, resequencing of 302 soybean lines revealed a separate cluster of diversity unique to Japan and Korea indicating a separate domestication event (Z. Zhou et al., 2015), which is supported by domestication‐associated SNPs detected only in Japanese lines (Jeong et al., 2019). The presence/absence of specific genes associated with the clusters presented here provide genic markers associated with this diversity (Supplemental Figure S4).

Domestication from wild soybean to cultivated soybean and subsequent selective breeding decreased nucleotide diversity, with the loss of the majority of the rare alleles and more than half of the genetic diversity (Hyten et al., 2006; Z. Zhou et al., 2015), and collectively only 17 landraces account for 86% of the North American genepool (Gizlice et al., 1993; Rincker et al., 2014). Soybean cultivars with a broad range of maturity and flowering time traits have been developed (Valliyodan et al., 2016; Z. Zhou et al., 2015), and an understanding of the genomic changes that occurred during domestication and breeding may assist in the identification of new alleles or genes to support future soybean breeding.

**FIGURE 2:**
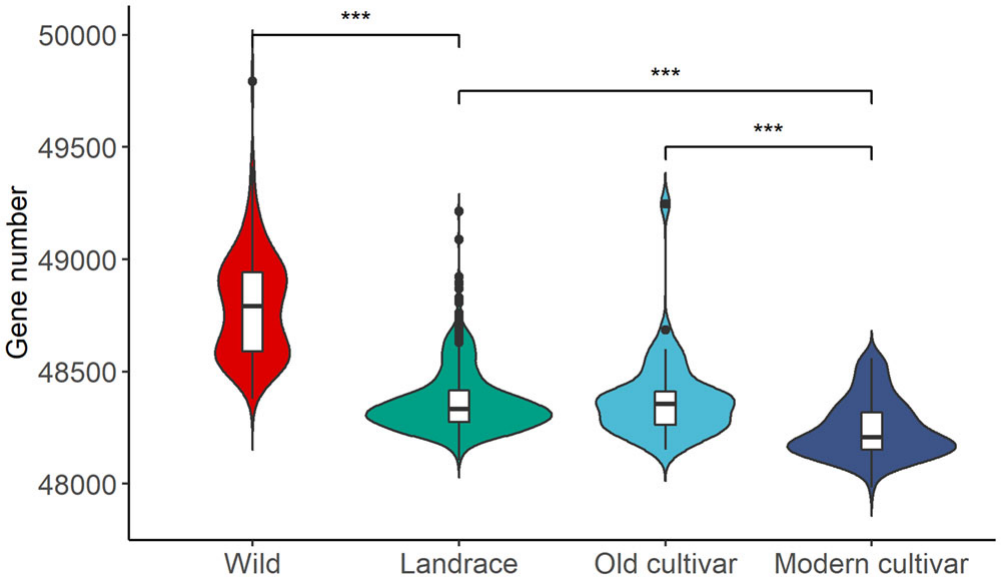
Violin plots showing gene abundance for the wild (*G. soja*), landraces, old and modern cultivars. Significance differences between groups is indicated (***p < .005 )

Analyzing gene content across this diverse population demonstrated a significant reduction in average gene number per individual following domestication and during subsequent breeding, similar to what was observed in a previous tomato pangenome study (Gao et al., 2019). Wild soybean contains the greatest average number of genes (48,785 ± 237), with a reduction in domesticated landraces (48,371 ± 139) and further declines in old cultivars (48,350 ± 232) and modern cultivars (48,165 ± 55) (Figure 2 and Supplemental Tables S6–S9). The loss of genes reflects in an overall reduction in genome size, with modern cultivars having an estimated average genome size of 877 Mbp compared with 898 Mbp for wild soybean (Supplemental Figure S5, Supplemental Table S10). On a country‐by‐country basis, the U.S. lines have a lower average gene number (48,286) than the other four major countries, for example, China (48,361), Korea (48,390), Japan (48,371), and Russia (48,344) (Supplemental Figure S6), mostly due to reduced average gene number in modern cultivars, suggesting that gene loss has accelerated in recent U.S. breeding programs. We also observed a greater average gene number in northern (48,332) compared with southern (48,204) adapted U.S. cultivars (Supplemental Figure S7). In heterozygous grapevine (*Vitis vinifera* ssp. *Sativa* L.), hemizygous genes are associated with PAVs accumulated during domestication and breeding (Y. Zhou et al., 2019). We expect hemizygosity to correlate with heterozygosity and therefore investigated patterns of heterozygosity across *G. soja*, landraces, as well as old and modern cultivars. There was a statistically significant difference in heterozygosity between *G. soja* and old cultivars, *G. soja* and modern cultivars, landraces and old cultivars, and landraces and modern cultivars (*p* < .05). However, the loss of genes following domestication does not mirror the decline of heterozygosity with no statistical difference in heterozygosity between old and modern cultivars (Supplemental Figure S8).

**FIGURE 3:**
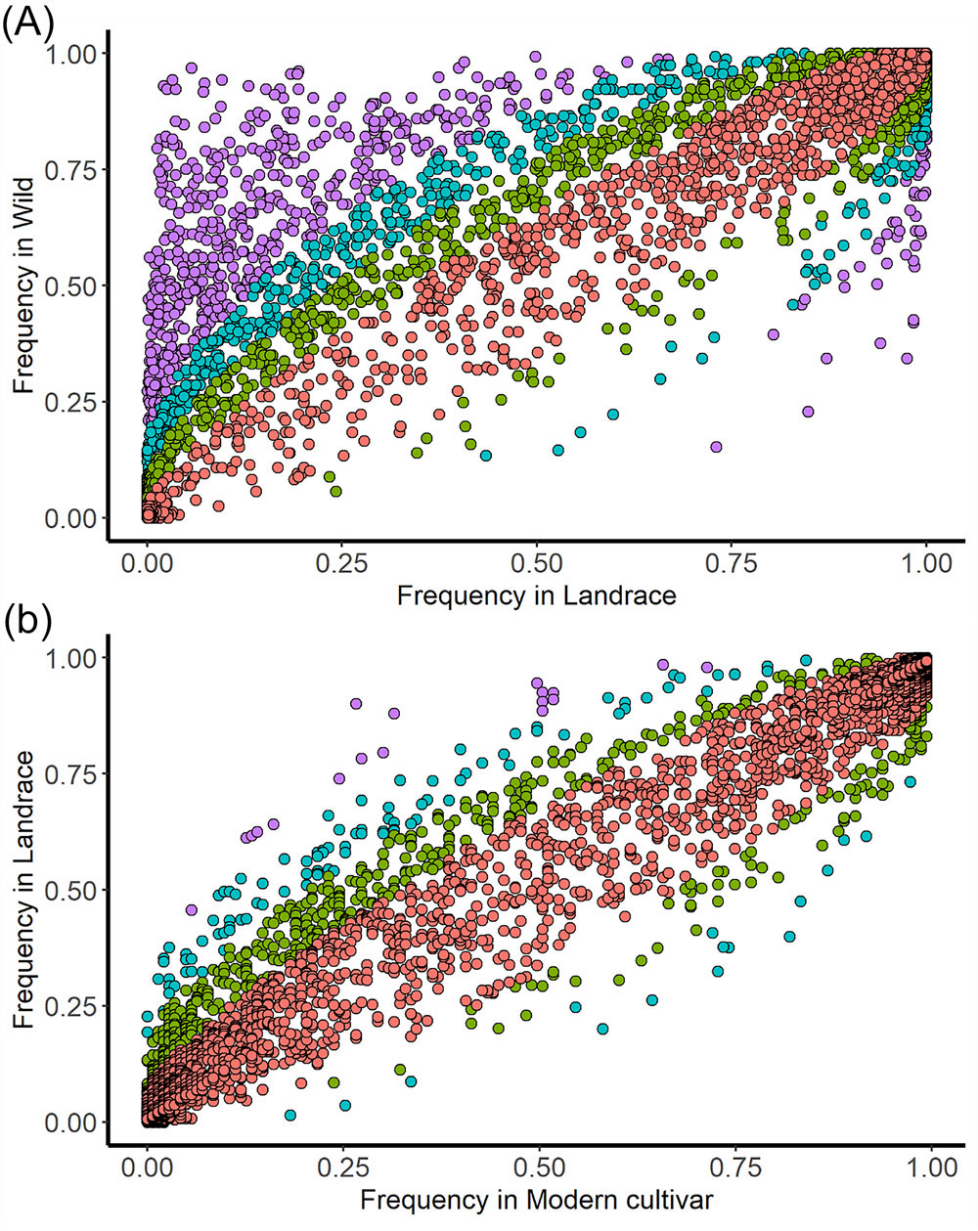
Comparison of gene frequency during soybean (a) domestication, and (b) breeding. Colors indicate *p*‐value, with purple (*p* < 1e‐20), blue (*p* > 1e‐10 ‐ ≤ 1e‐20), green (*p* > .01 ‐ ≤ 1e −10), and red (*p* < = .01)

The reduction in average gene numbers hides a more complex pattern of increases and decreases in the frequency of specific genes across the population. To identify gene PAV changes during soybean domestication, we compared gene frequencies between wild soybean and landraces (Figure 3). A total of 1,478 genes decreased in frequency following domestication, while 261 genes increased in frequency (Figure 3a, Supplemental Table S6–S7). Among the annotated genes with decreased frequency, 98 were associated with defense response, 88 were associated with protein kinase activity, 44 with oxidation‐reduction process, and 36 with response to salt process. Thirteen of the 98 defense response genes are colocated with disease resistance quantitative trait loci (QTL), including *Sclerotinia* resistance, Sclero 3‐g31 and Sclero 3‐g58 (Moellers et al., 2017), brown stem rot (BSR) resistance, BSR 1‐g2 (Chang et al., 2016), and *Phytophthora* resistance, Phytoph 2‐g1, Phytoph 2‐g6 and Phytoph 2‐g17 (Qin et al., 2017; Supplemental Table S11).

Genes associated with pubescence color were affected by domestication (Han et al., 2016), and the pubescence color gene GlymaLee.12G119700 shows a reduction in gene frequency from 79% in wild soybean to 38% in the landraces. Flowering time is also under strong selection during domestication, breeding, and adaptation, and several flowering related genes, including FRIGIDA‐like protein 4a, demonstrate a reduction in frequency during domestication (Supplemental Table S6). While fewer genes increase in frequency following domestication, they include 22 disease resistance genes and 10 salt stress tolerance genes suggesting selection for these traits (Supplemental Table S7).

Early breeding efforts developed cultivars suitable for North American production systems, and as soybean production increased, the breeding programs focused on yield improvement and disease resistance traits. Breeding for yield over the last 60 yr has had no major influence on seed protein composition, possibly because of limited genetic diversity among the parental lines (Mahmoud et al., 2006). The average number of genes per individual declined during breeding (Figure 2), and we observed a decrease in frequency for 483 genes, while 100 genes increased in frequency during the transition from landrace to modern cultivar (Figure 3b). Among the genes that reduce in frequency, 49 were associated with defense response, 36 with signal transduction, 15 with oxidation‐reduction process, and 7 with response to auxin stimulus (Supplemental Table S8). Genes that reduce in frequency during breeding are associated with QTL for plant architecture and seed composition traits, including three genes under the Shoot Fe 1‐g20, one under the Leaf carotenoid content QTL 1‐g13.4, and another 5 associated with seed composition and yield QTL (Supplemental Table S12). Genes that increase in frequency during breeding are mainly associated with flowering time, seed composition, and stress tolerance traits (both disease resistance and abiotic stress), though genes encoding several auxin responsive proteins that share maturity and seed composition functions also increased frequency during breeding (Supplemental Table S9).

Comparing cultivars from the five most represented countries (Russia, China, Japan, United States, Korea) identified 16 genes that increased in frequency and 64 genes that decreased in frequency in U.S. cultivars compared with each of the four other countries (Supplemental Table S13–S14). Several of the genes that reduce in frequency encode disease resistance genes, including UWASoyPan03234, a Leaf Rust 10 disease‐resistance locus receptor‐like gene; UWASoyPan03449, a TMV resistance protein N isoform X3; and UWASoyPan05034, a disease resistance RML1A‐like gene. More detailed comparison of northern and southern U.S. lines identified 27 genes that have a lower frequency and 8 genes that have a higher frequency in southern cultivars (Supplemental Table S15). Of the 27 genes, 3 show similarity to transcription factors, while 5 show similarity to disease resistance genes. The eight genes that increased in frequency in southern adapted cultivars include *ZPR1*, which encodes a clock‐associated zinc finger protein required for circadian‐regulated gene expression in plants (Kiełbowicz‐Matuk et al., 2017 ; J. Li et al., 2013), and so may play a role in adaptation. While we have sequenced a large and diverse collection of germplasm, we only have a limited insight into local diversity and selection, and the sequencing of additional lines may reveal a more complete picture of genome variation due to local soybean breeding efforts.

Studies have shown that domestication from wild soybean to landraces resulted in a reduction in genetic diversity and the loss of more than 81% of rare alleles (Hyten et al., 2006; Z. Zhou et al., 2015). Early North American landraces have low genetic diversity compared to Chinese lines (Y. Li et al., 2008), and southern elite cultivars are less diverse compared to the ancestral U.S. cultivar pool (Kisha et al., 1998; Thompson & Nelson, 1998). Here, we annotated 13,039,091 high‐quality SNP loci across the 1,110 individuals and called 14,285,049,178 genotypes. The nucleotide diversity (π) of wild soybeans (3.75 × 10^−3^) was higher than landraces (2.12 × 10^−3^), old cultivars (2.11 × 10^−3^), and modern cultivars (1.48 × 10^−3^), reflecting the loss of diversity during domestication and breeding. These values are similar to previous observations in U.S. (Hyten et al., 2006; Valliyodan et al., 2016) and Chinese soybean lines (Z. Zhou et al., 2015), suggesting that U.S. and Chinese lines show similar nucleotide diversity and similar loss of diversity during domestication and subsequent breeding.

**FIGURE 4:**
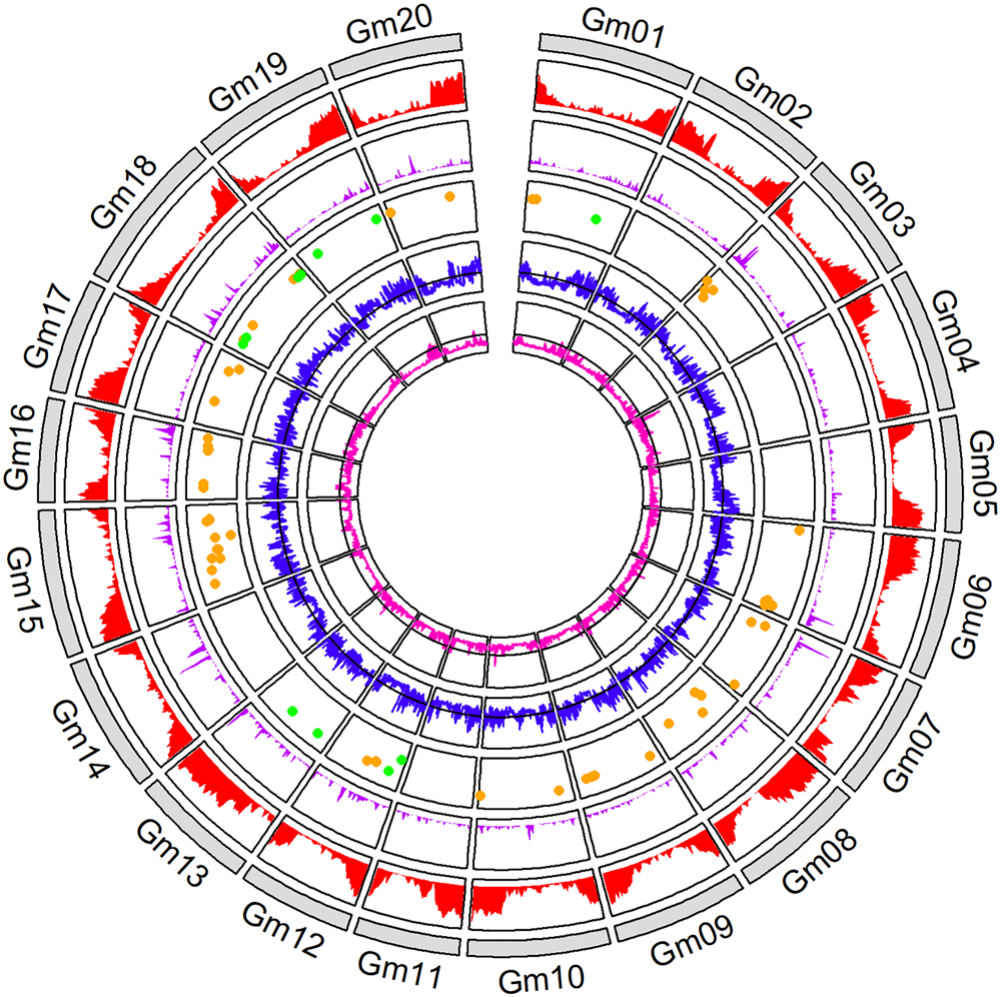
Circos plot showing genetic diversity and signals of selection between landraces and modern cultivars. From outer‐most track to innermost track: (a) gene density; (b) dispensable gene density; (c) genes increased and decreased between landraces and modern cultivars (false discovery rate (FDR)‐adjusted *p* < 0.05, orange: genes increased in frequency in modern cultivars, green: genes decreased in frequency in modern cultivars) (y‐position assigned to avoid overlapping points); (d) Tajima's D between landraces and modern cultivars (black line: D = 0); and (e) Fixation Index (FST) between landraces and modern cultivars (black line: fixation index [FST] = 0.15)

The average distance over which LD decays to half of its maximum value was substantially shorter in wild soybean than landraces and old and modern cultivars, which shows similar trend to previous studies (Hyten et al., 2006; Valliyodan et al., 2016 ; Z. Zhou et al., 2015; Supplemental Figure S9 and Supplemental Table S16). We searched for selective sweeps during domestication and breeding and identified 110 genomic regions with signatures of domestication‐selective sweeps harboring 1,266 protein‐coding genes. We also identified 86 genomic regions with signatures of breeding‐selective sweeps harboring 1,434 protein‐coding genes (Supplemental Table S17–S18, Figure 4). Among the genes located within the domestication‐selective sweeps, 51 genes are dispensable, with a probable receptor‐like protein kinase GlymaLee.05G082900 and a L‐10 interacting MYB domain‐containing protein GlymaLee.05G083000 showing a significant decrease in frequency during domestication. In total, 55 genes are dispensable among the genes located within selective sweep regions during breeding. Four of these dispensable genes—including omega‐6 fatty acid desaturase GlymaLee.15G174100, a phosphate transporter GlymaLee.15G174200, a nonannotated gene GlymaLee.17G150300, and a GEM‐like protein GlymaLee.20G073500—significantly increased in frequency during breeding, and only one gene GlymaLee.19G173000, encoding a transmembrane protein, significantly decreased in frequency.

Domestication selection sweeps on chromosome Gm20 (6.9–12 Mb), overlapping with the seed protein QTL were previously detected in other reported domestication‐related QTL regions (Grant et al., 2010; Nichols et al., 2006). We also found a breeding‐related selective sweep region on Gm20 (36.1–37.2 Mb), which overlapped with the reported Seed yield 31–38, seed oil and seed protein QTL region (Grant et al., 2010; Hacisalihoglu et al., 2018). These results show that selective sweeps acted on different QTL regions during domestication and breeding.

Calculation of the divergence index value (FST) between different groups identified genomic regions associated with domestication and subsequent breeding. The largest differences of FST were observed during domestication, with a mean weighted value of 0.215 (Supplemental Table S16), compared with 0.06 between landraces and modern cultivars. These results are consistent with previous studies showing that wild soybean contains the most diverse gene pools and that selective sweeps are stronger during domestication than during breeding (Hyten et al., 2006; Song et al., 2020; Valliyodan et al., 2016 ; Z. Zhou et al., 2015).

In this study, we have examined changes in the frequency of dispensable genes during domestication and breeding, providing information that will assist the production of improved cultivars. The reduction in average gene number and genome size during breeding was unexpected and raises several questions. If breeders are selecting for gene absence, then selection can only occur for the relatively small proportion of genes that show PAV. Further analysis may identify candidate core genes that, if deleted using tools such as genome editing, could further improve this important crop. Moreover, this pangenome, along with the publicly available USDA Soybean Germplasm Collection, provides a valuable resource to design more efficient and targeted molecular breeding strategies for soybean improvement.

## AUTHOR CONTRIBUTIONS

Philipp E. Bayer, Babu Valliyodan, and Haifei Hu contributed equally.

## CONFLICT OF INTEREST

The authors declare no conflict of interest.

## Supporting information


**Supplemental Figure S1**. Geographic distribution of soybean accessions with clear origin record.
**Supplemental Figure S2. a)**: Principal component analysis based on gene PAVs. **b)**: Principal component analysis based on SNPs. Red: wild. Green: landrace. Light blue: old cultivar.
**Supplemental Figure S3**. Phylogenetic tree inferred from whole‐genome gene PAVs. Branches and label ranges are colored by group membership.
**Supplemental Figure S4**. Landscape of dispensable gene PAVs. Soybean accessions were clustered based on the PAV binary matrix.
**Supplemental Figure S5**. Estimates of genome size differences between the four soybean groups, showing that modern cultivars have statistically significantly smaller genome sizes (**p < .005, average: −20.5 Mbp in modern cultivars compared with old cultivars).
**Supplemental Figure S6**. Gene number differences between the five origin countries with the most‐sampled individuals, showing that the United States has a statistically significant difference in the mean of gene numbers (***p < .0005).
**Supplemental Figure S7**. Gene frequency comparison between Northern and Southern adapted cultivars for U.S. soybeans.
**Supplemental Figure S8**. Loss of heterozygosity as measured using the inbreeding coefficent *F* from *G. soja* to landraces, old and modern cultivars (NS, not significant, *p < .05, **p < .005, Wilcoxon Rank Sum test).
**Supplemental Figure S9**. The average distance over which LD decays to half of its maximum value was substantially longer in wild soybean than landraces, old and modern cultivars.

Supplemental Table S3. Summary of gene PAVs
Supplemental Table S4. Gene count and percentage of core gene and dispensable gene in different group
**Supplemental Table S5. GO terms enriched in dispensable genes**.
**Supplemental Table S6. Genes that significantly decreased in frequency during domestication**.
Supplemental Table S7. Genes that significantly increased in frequency during domestication
**Supplemental Table S8. Genes that significantly decreased in frequency during breeding**.
**Supplemental Table S9. Genes that significantly increased in frequency during breeding**.
Supplemental Table S10. Genome size estimates for the four groups
Supplemental Table S11. QTL information for the genes that significantly decreased in frequency during domestication (NA, not applicable)
Supplemental Table S12. QTL information for the genes that significantly decreased in frequency during breeding. (NA, not applicable)
Supplemental Table S13. Genes that increased in frequency in U.S. cultivars compared to the four other most represented countries (Russia, China, Japan, and Korea). (NS, not determined)
**Supplemental Table S14. Genes that decreased in frequency in U.S. cultivars compared to the four other most represented countries (Russia, China, Japan, and Korea). (ND, not determined)**.
Supplemental Table S15. Genes that significantly change in frequency in Southern U.S. cultivars compared to Northern U.S. cultivars. (ND, not determined)
**Supplemental Table S16. Genetic diversity between different groups indicated by average FST values and nucleotide diversity**.
Supplemental Table S17. Genes under domestication related selective sweeps. (NA, not applicable)
Supplemental Table S18. Genes under breeding related selective sweeps. (NA, not applicable)
