# Supplementary material for: Sequencing the USDA core soybean collection reveals gene loss during domestication and breeding
Source: Plant Genome. 2021 Jun 24;15(1):e20109. doi: 10.1002/tpg2.20109 (PMC12807262; doi:10.1002/tpg2.20109)
Supplement: Supplementary file 1 [file TPG2-15-e20109-s002.pdf]

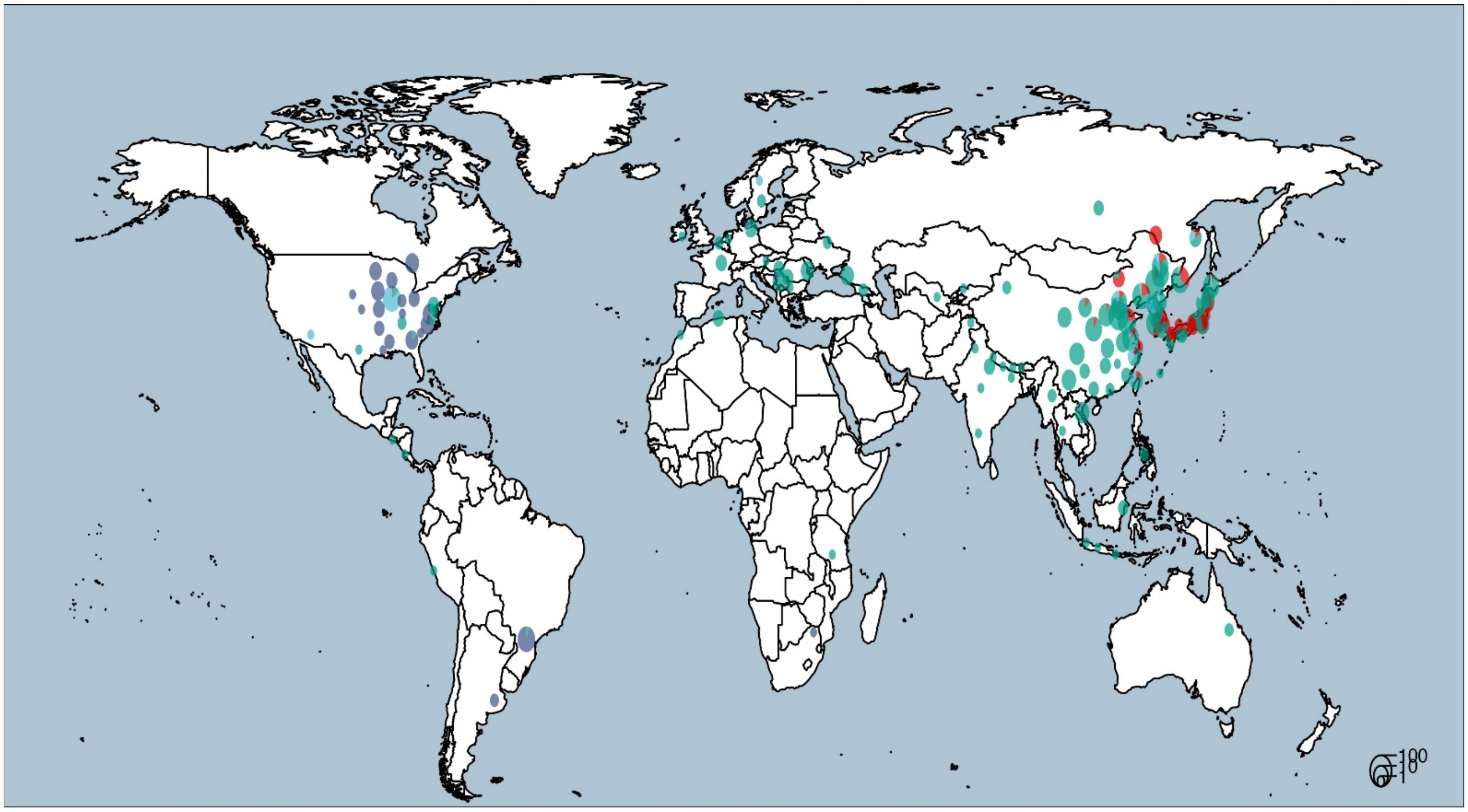

**Supplemental Figure S1.** Geographic distribution of soybean accessions with clear origin record. The number of accessions collected from each region is indicated by the size of pie, and the ratio of wild soybean (red), landrace (green), old cultivar (light blue) and modern cultivar (purple) for each region is shown in the pie.

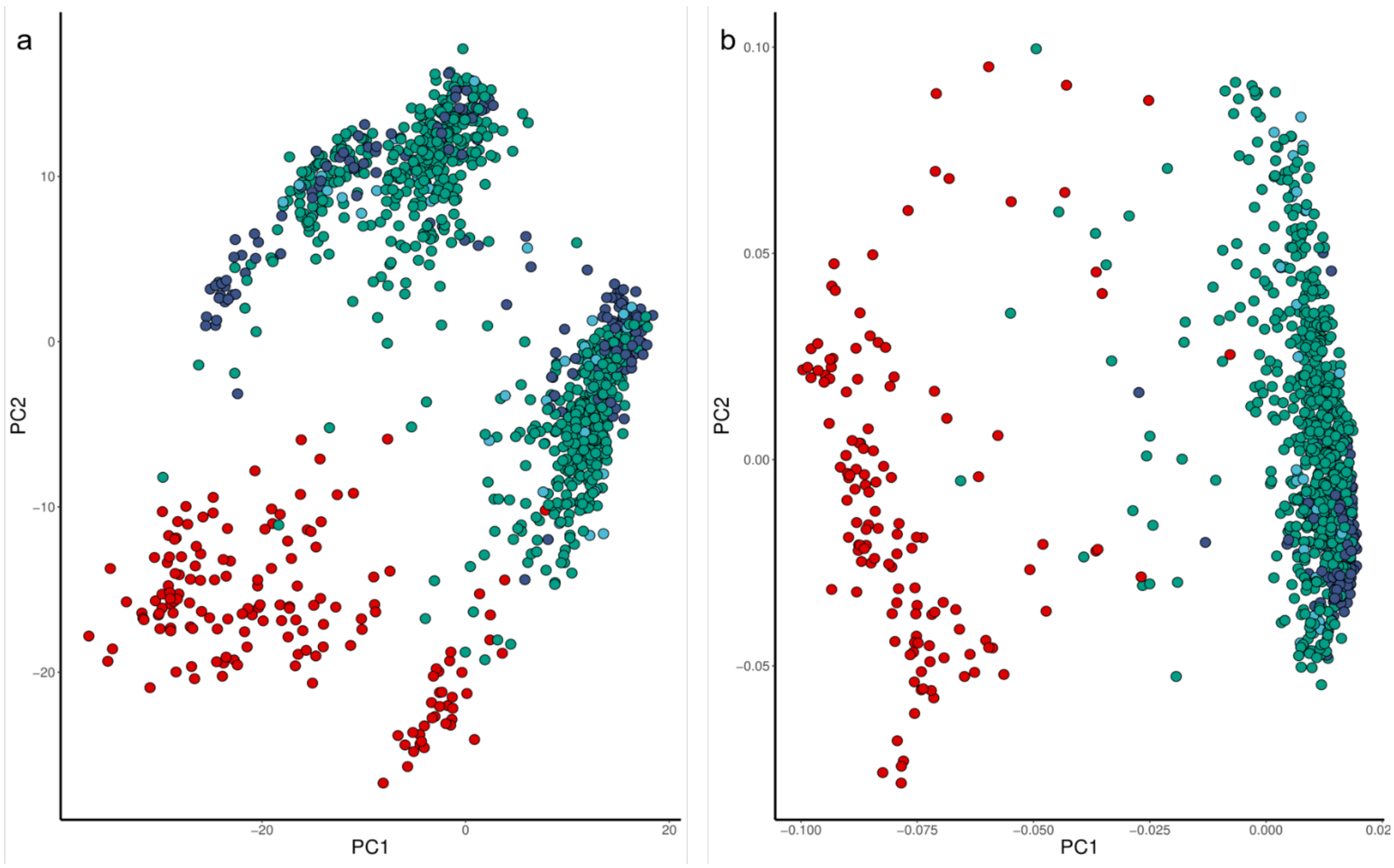


**Supplemental Figure S2. a):** Principal component analysis based on gene PAVs. **b):** Principal component analysis based on SNPs. Red: wild. Green: landrace. Light blue: old cultivar. Purple: modern cultivar.

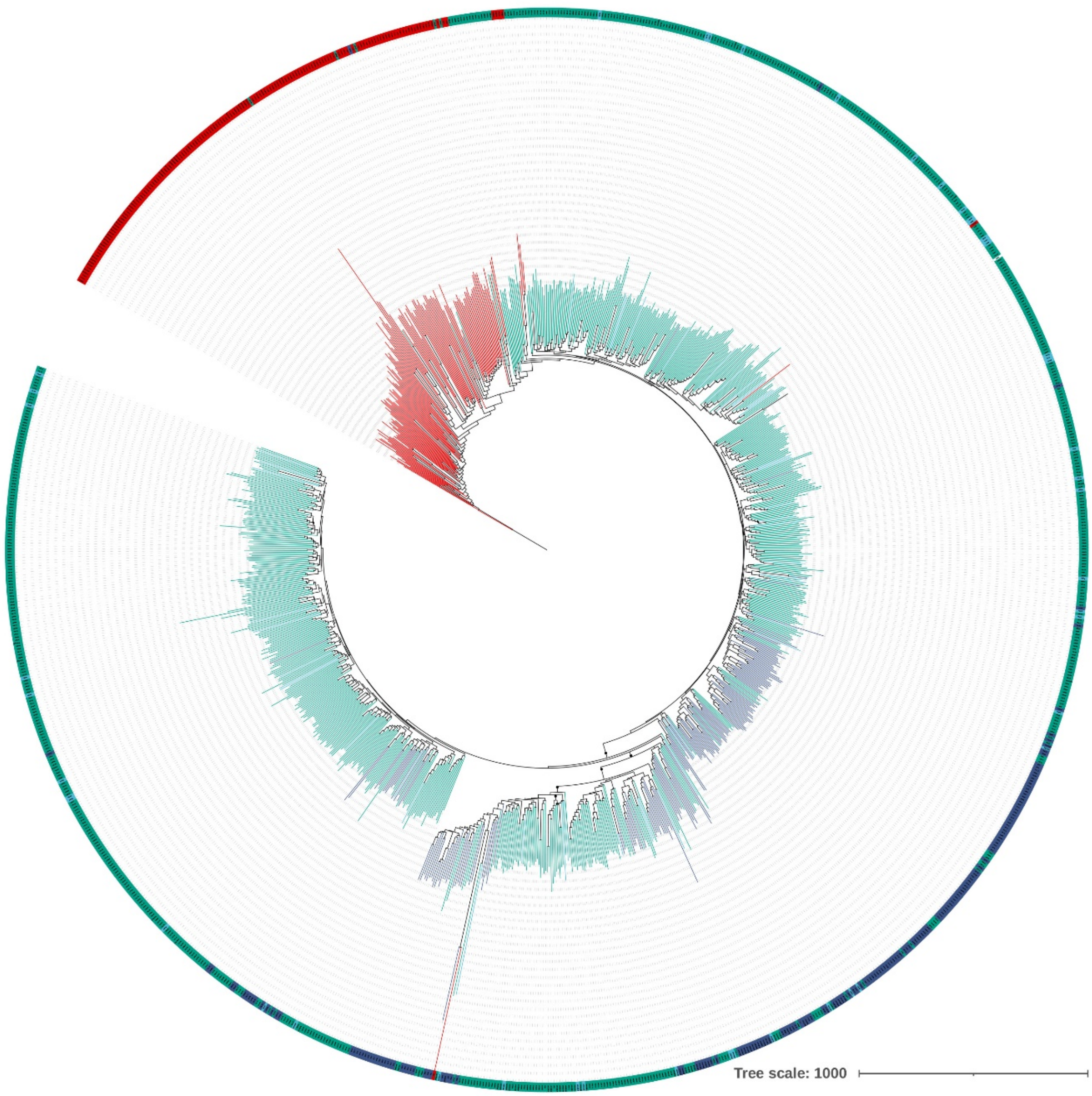


**Supplemental Figure S3.** Phylogenetic tree inferred from whole-genome gene PAVs. Branches and label ranges are colored by group membership. Red: wild. Green: landrace. Light blue: old cultivar. Purple: modern cultivar.

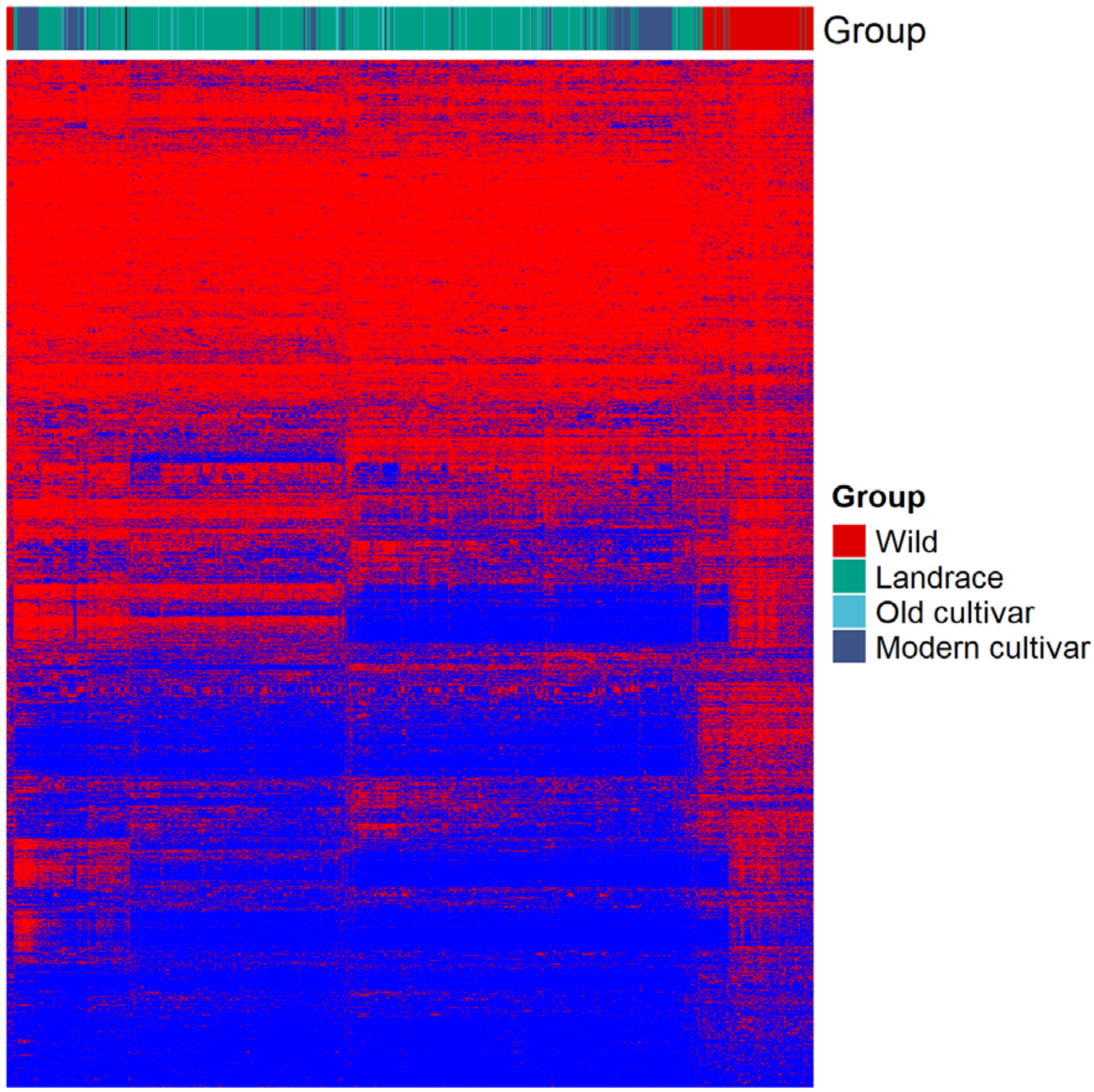


**Supplemental Figure S4.** Landscape of dispensable gene PAVs. Soybean accessions were clustered based on the PAV binary matrix. Red represents the absent gene and Blue represents the present gene.

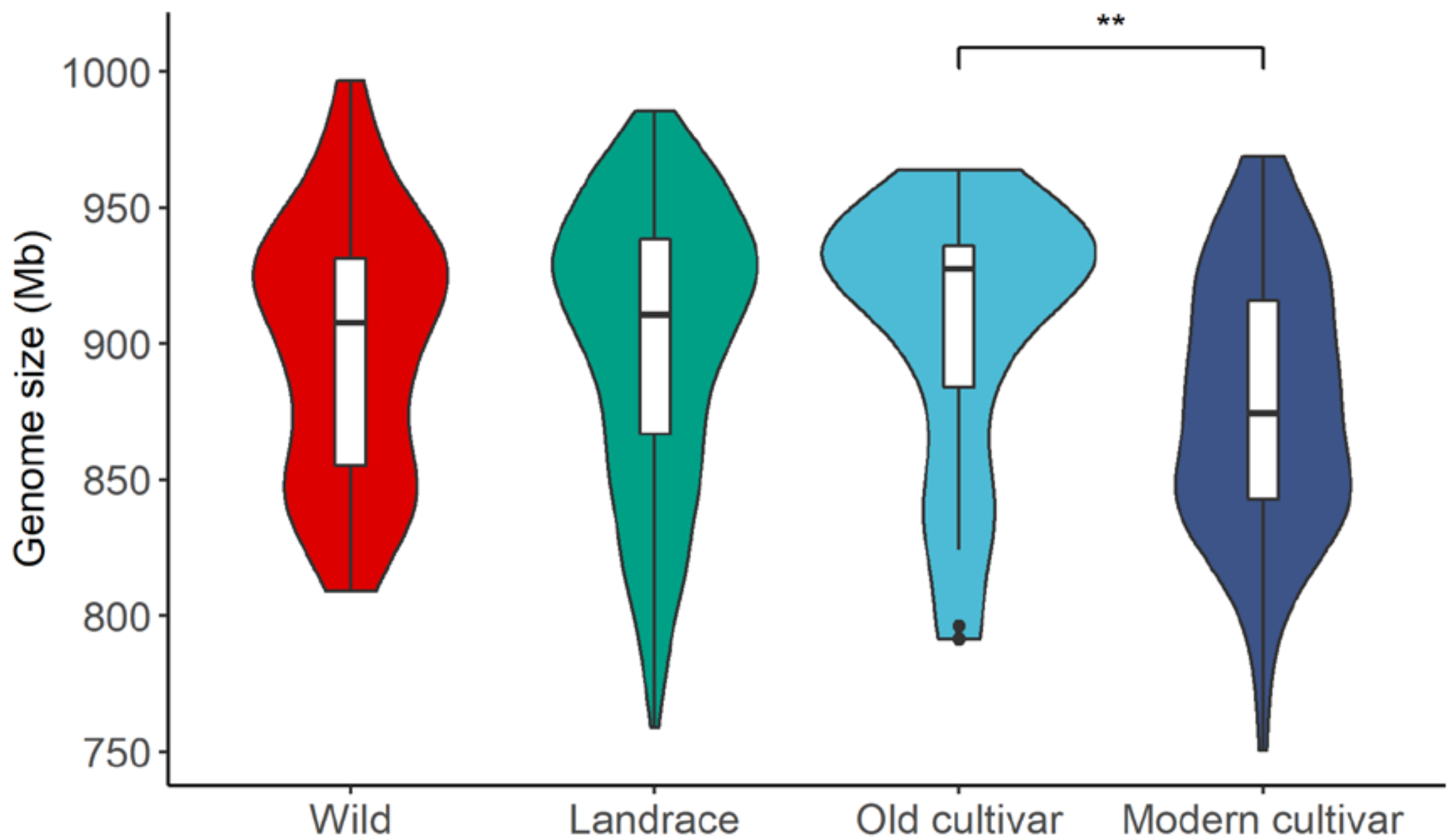


**Supplemental Figure S5.** Estimates of genome size differences between the four soybean groups, showing that modern cultivars have statistically significantly smaller genome sizes (**: $p < 0.005$, average: -20.5 Mbp in modern cultivars compared with old cultivars)

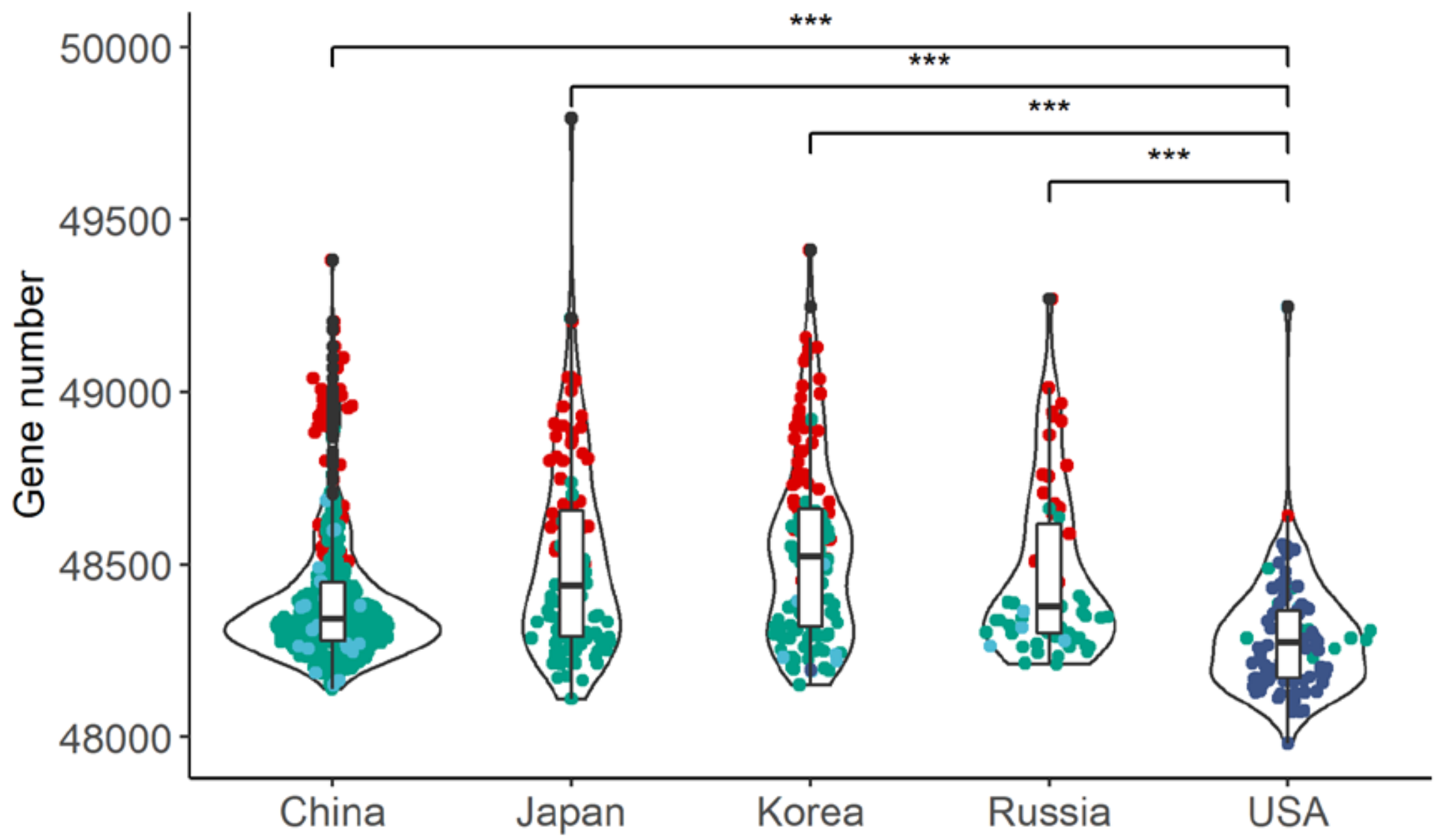


**Supplemental Figure S6.** Gene number differences between the five origin countries with the most-sampled individuals, showing that the US has a statistically significant difference in the mean of gene numbers (***: p<0.0005). Points are colored by group membership. Red: wild-type. Green: landrace. Light blue: old cultivar. Purple: modern cultivar.

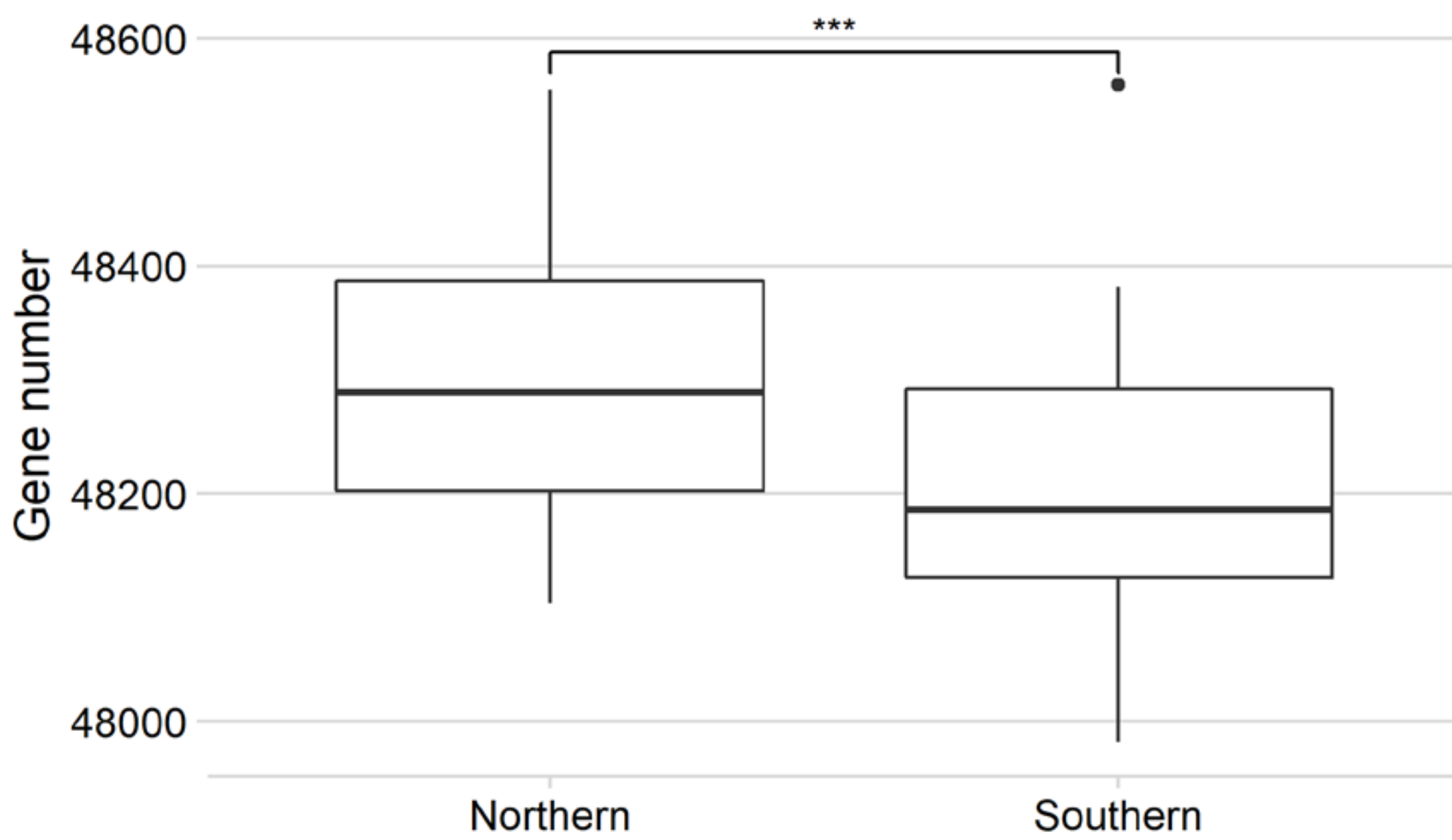


**Supplemental Figure S7.** Gene frequency comparison between Northern and Southern adapted cultivars for US soybeans. *** = $p < 0.0005$.

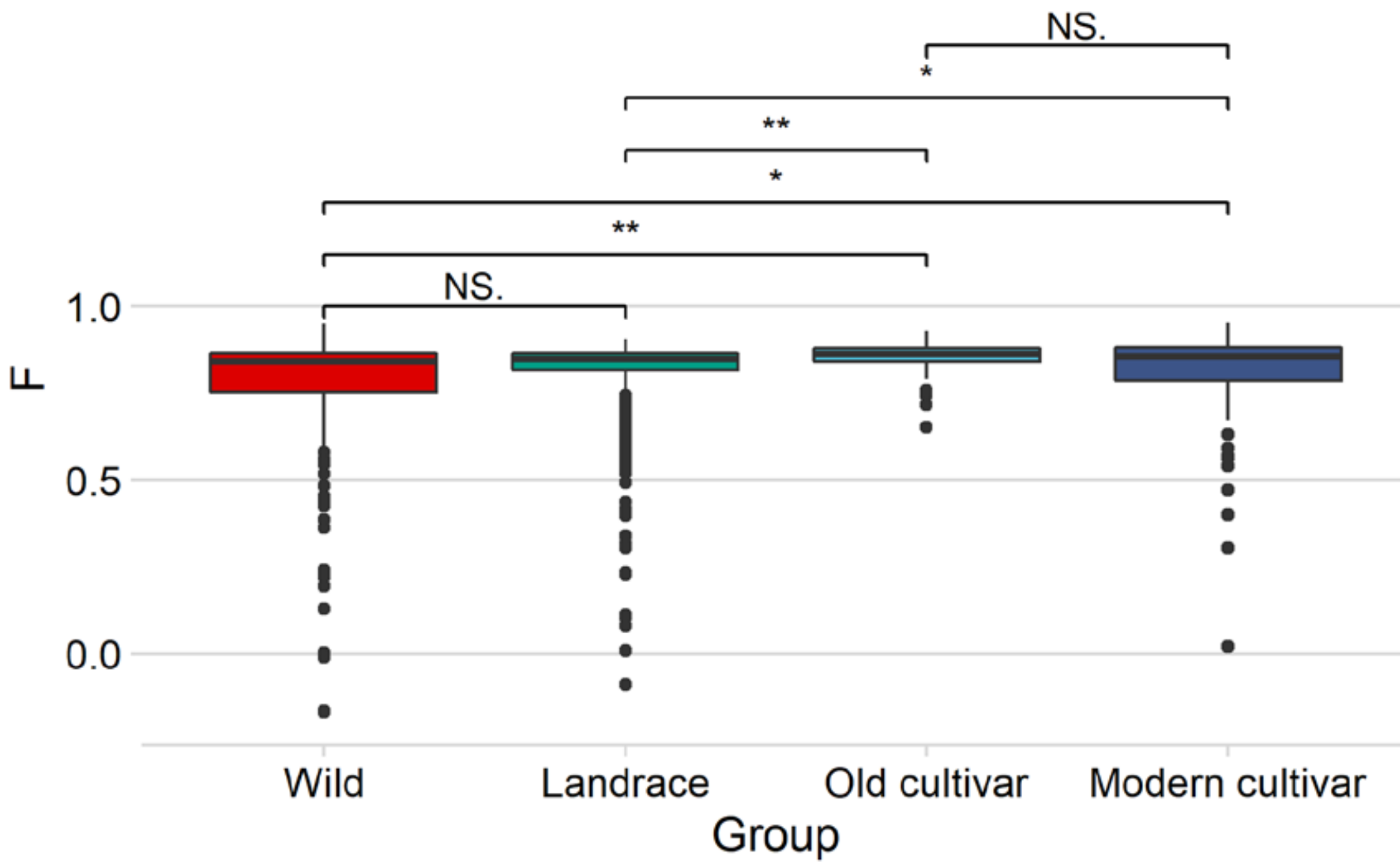


**Supplemental Figure S8**. Loss of heterozygosity as measured using the inbreeding coefficent *F* from *G. soja* to landraces, old and modern cultivars (NS, not significant, *, $p < 0.05$, **, $p< 0.005$, Wilcoxon Rank Sum test).

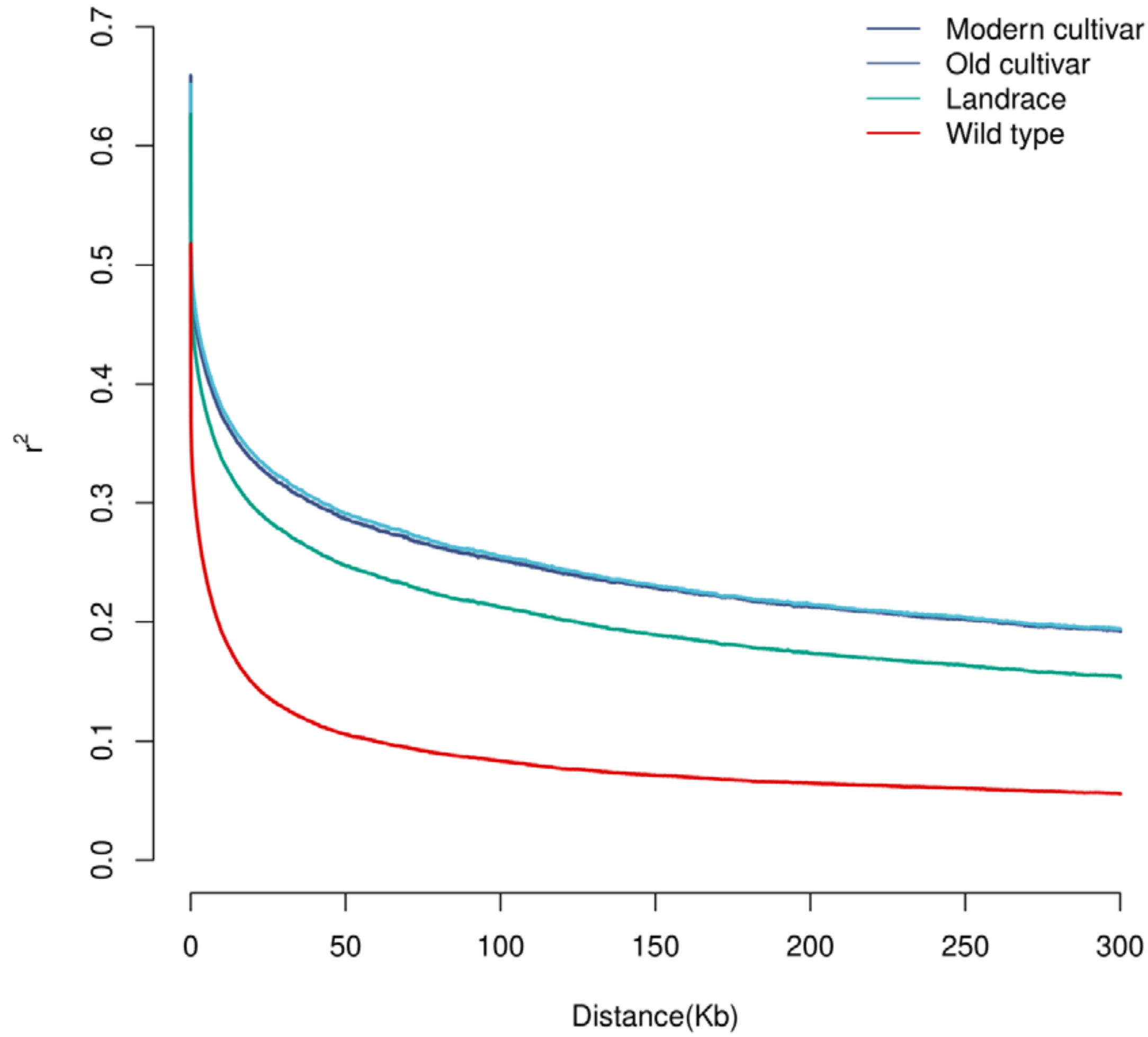


**Supplemental Figure S9**. The average distance over which LD decays to half of its maximum value was substantially longer in wild soybean than landraces, old and modern cultivars.
